# Application of acute pre-exercise partial-body cryotherapy promotes jump performance, salivary α-amylase and athlete readiness

**DOI:** 10.5114/biolsport.2022.107019

**Published:** 2021-07-15

**Authors:** Emily M. Partridge, Julie Cooke, Andrew J. McKune, David B. Pyne

**Affiliations:** 1 Research Institute for Sport and Exercise Science (UCRISE), University of Canberra, Bruce, ACT, Australia; 2 Faculty of Health, University of Canberra, Bruce, ACT, Australia; 3 Discipline of Biokinetics, Exercise and Leisure Sciences, School of Health Sciences, University of KwaZulu-Natal, Durban, KwaZulu-Natal, South Africa

**Keywords:** Resistance exercise, Post-activation potentiation, Neuromuscular performance, Physiology biomarkers, Autonomic nervous system

## Abstract

This study aimed to evaluate the application of a single pre-exercise bout of partial-body cryotherapy (PBC) to augment jump performance, salivary biomarkers and self-reported performance readiness. Twelve male rugby union players (age 20.7 ± 3.2 yr; body mass 93.1 ± 13.9 kg; mean ± SD) were exposed to PBC for 3 min at –140°C or control condition prior to a pre-post series of loaded countermovement jumps (CMJ), salivary biomarker samples and performance readiness questionnaires. PBC elicited a moderately greater improvement in CMJ velocity of +4.7 ± 3.5% (mean ± 90% confidence limits) from baseline to 15 min in comparison with a -1.9 ± 4.8% mean difference in the control condition. The mean change in concentration of salivary α-amylase at 15 min was substantially increased by +131 ± 109% after PBC exposure, compared to a -4.2 ± 42% decrease in the control. Salivary testosterone concentrations were unclear at all timepoints in both the PBC and control interventions. Self-reported perceptions of overall performance readiness indicated small to moderate increases in mental fatigue, mood, muscle soreness and overall questionnaire score after PBC compared to control with a higher score more favourable for performance. The application of pre-exercise PBC can elicit favourable outcomes in controlled physical performance tests and holds promise to be applied to training or competition settings.

## INTRODUCTION

The hours preceding competition are utilised in various ways to prepare athletes, so they perform at their peak both psychologically and physically. A well-structured warm-up alongside strategies of post-activation potentiation, ischemic preconditioning, morning exercise or hormonal priming can lead to performance benefits in competition [1]. The importance of pre-competition preparation and warm-up routines for optimal performance is widely acknowledged by coaches and athletes alike [2]. Warm-ups typically involve both active and passive techniques that can be highly individualised based on personal and team preferences. With modern technological advances, a range of strategies are available for improving passive warm-ups in conjunction with dynamic protocols to better prepare athletes for competition.

Partial-body cryotherapy (PBC) has recently gained popularity as a means for promoting well-being and recovery for athletes [3, 4]. PBC is a cytotherapeutic modality which safely exposes users to temperatures of –110°C to –140°C for periods of 1–3 min [5]. PBC has been thoroughly investigated as a recovery protocol involving either an acute exposure or multiple bouts over periods of 5–10 days [6]. However, nearly all studies used PBC post-exercise [5]. PBC has previously yielded increased stimulation of the autonomic nervous system [7], sleep quality, and mood and well-being [8], as well as skill-based performance outcomes in some high-level athletes [9]. A single session of cryotherapy (30 sec at –60°C and 120 seconds at –120°C) can increase testosterone concentrations in elite soccer players for up 24 h post-exposure [10]. These effects could potentially have direct or indirect improvements on athletic performance.

Despite the plethora of post-exercise studies on athletic populations, few studies have investigated the immediate effects of acute pre-exercise PBC on athletic performance. The majority of studies investigating pre-exercise cryotherapy exposure have utilised whole-body cryotherapy (WBC), which is a similar exposure method but differs due to the users’ head being in the chamber. Of the research using PBC or WBC pre-exercise, most studies investigated the effects on anti-inflammatory or oxidative stress recovery from submaximal and high-intensity exercise, with favourable results conducive to performance outcomes [11, 12]. Pre-exercise WBC has elicited improvements in sit-and-reach scores by ~2 cm in men and ~3 cm in women compared to the control conditions [13]. Furthermore, PBC exposure immediately prior to maximal isometric grip strength tests elicited an improvement by 5% in women and 2% in men compared to the control [14]. This effect has been attributed to a priming response by inducing neuromuscular facilitation, and increasing the sympathetic nervous response to intense cold exposure [15]. Salivary α-amylase is correlated moderately (r = 0.30–0.66) with increases in blood catecholamines specifically, dopamine, epinephrine, and norepinephrine in both healthy men and elite female archery athletes [16]. Acute cryotherapy exposure has been shown to substantially increase the concentration of plasma norepinephrine [7, 17]. Given the physiological benefits of PBC exposure, coaches, athletes and practitioners alike could benefit from further research addressing the immediate effects on commonly utilised performance benchmark testing protocols.

This study aimed to quantify whether a single bout of PBC acutely influences jump performance and associated physiological and perceptual factors, in a cohort of well-trained rugby union players. We expected that an acute 3 min PBC exposure could elicit a beneficial potentiation and neuromuscular facilitation effect. We also expected that salivary biomarkers α-amylase (as an indirect marker of blood catecholamine concentration) and testosterone would be higher than under control conditions. Self-reported psychological variables important for game or training performance were expected to mirror this increase. To the best of our knowledge, this is the first study to analyse the immediate effects of acute pre-exercise PBC on countermovement jump (CMJ) performance.

## MATERIALS AND METHODS

### Participants

A group of 12 male state-level rugby union players (age: 20.8 ± 3.1 yr; body mass: 93.1 ± 13.0 kg; height: 1.82 ± 0.05 m; mean ± SD) were recruited to participate in the research. Each of the participants had been training for at least 1 yr, prior involving 5 ± 1 sessions per week (including resistance, rugby-specific and recovery sessions). The research was conducted during the in-season period with all data collection completed between 1–6 pm, 3 days after scheduled weekend games. After explanation of benefits, experimental risks and procedures involved, participants provided written informed consent. Approval from the Human Ethics Committee at the University of Canberra was obtained (HREC–0213).

The rugby team physician was consulted for any contraindications that may exclude a participant from the study. Contraindications included: asthma, Reynaud’s phenomenon, hypothyroidism, cardiac disease or injuries preventing physical activity. The health and medical screening questionnaire indicated that no participants had any contraindications that would impact their ability to complete the study.

### Design

A test-retest protocol using a randomised, counterbalanced crossover design was employed to investigate the acute effects of a single 3 min PBC exposure in a simulated 3 h warm-up period on CMJ performance. The participants completed both a PBC intervention and a control trial in a randomised and counterbalanced order, with a two week washout period between each trial, allowing for any residual effects of the PBC to dissipate [18]. The control condition involved the participants completing all testing procedures without the PBC exposure intervention.

### Protocol

**FIG. 1:**
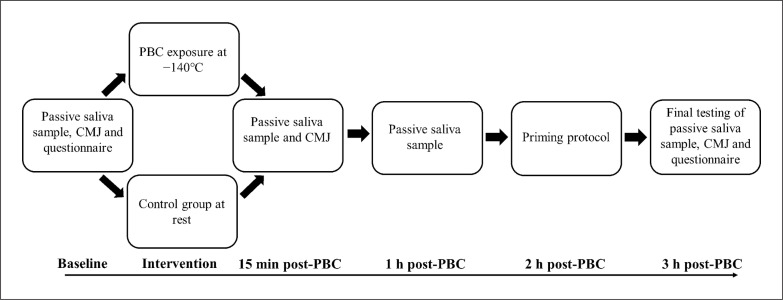
Design of experimental protocol and variables collected at each time point of the 3 h post-partial-body cryotherapy exposure period. CMJ = countermovment jump and PBC = partial-body cryotherapy.

Each participant’s body mass (kg) was recorded immediately upon arrival at the testing facility (Figure 1). All participants were instructed not to consume any food or fluid 30 min prior to providing a saliva sample. Saliva was collected using a sterilised cryovial, and participants were given a maximum of 5 min to attain the 1 mL volume required. If the participant did not achieve the required volume in 5 min, they rested for an additional 15 min and the process repeated. Saliva samples were stored in a freezer immediately upon collection. Saliva was assayed for testosterone and α-amylase (sAA) concentrations using commercial enzyme immunoassay kits (Salimetrics LLC, USA). α-amylase had an intra- and inter-assay variability of 3.6% and 4.1% respectively, and testosterone 8.1% and 4.9% respectively.

Participants completed a customised readiness to perform questionnaire, which listed variables of mental fatigue, sleep quality, muscle soreness, stress and aggression on a 5-point Likert scale in 1-point increments, with a higher number for each variable deemed more desirable for optimal performance. The questionnaire has previously been validated in research [19–21]. Questionnaires were completed in isolation to avoid any bias occurring.

Jump performance was recorded in mean velocity (m·s^-1^) and mean power (W) through CMJ tests with a load of 20 kg. The CMJ has a very high test-retest reliability of lower-body strength with a correlation of r = 0.97 and a typical error of 4% [22]. A GymAware (Kinetic Performance Technology, Canberra, Australia) tether was connected to a barbell and placed directly underneath the bar to avoid any angle of displacement away from vertical alignment. Each jump was encouraged to be a maximal effort with feet off the ground and an emphasis on the change of direction from the lowest point of the squat. Participants completed three warm-up jumps, followed by two maximal efforts with the mean power and velocity calculated as the criterion score.

The PBC protocol involved the participant standing in a JUKA Low-Temperature Cryo Sauna (Model 0104-1) for 3 min at –140˚C ± -30˚C with a lid placed at shoulder level and the head positioned above the chamber. Participants entered the chamber wearing woollen boots, socks, shorts and gloves. The internal Cryo Sauna temperature was recorded in 30 s intervals via a temperature sensor located at the bottom of the chamber. For the control condition, participants rested in a thermoneutral environment of 22.0 ± 0.9°C. Following the PBC exposure or control condition, the participants provided a saliva sample 15 min after exiting the chamber, then a CMJ measure following the same protocol performed previously. One hour after PBC exposure or control condition, the same procedure for a saliva sample was repeated.

Post-activation potentiation (PAP) in the form of weighted priming jumps was conducted 2 h post-PBC. This process was completed to simulate a controlled warm-up prior to the final maximal effort jumps. The participants performed four sets of three repetition squat jumps with a barbell weight totally to 0.4 × body mass, as previously defined in research [21]. Participants were encouraged to jump off the ground with maximal effort. After priming, no further physical activity or dynamic stretches were permitted. At 15 min prior to the priming exercises, a final saliva sample was provided. Participants completed a second wellness and performance readiness questionnaire 5 min before final performance testing. At 3 h post-PBC exposure, the final performance measures were recorded using the three-jump warm-up and two maximal efforts with the 20 kg barbell.

### Statistical Analyses

Sample size estimation using G*Power software (v3.1.9.4 a priori power analysis with ANOVA repeated measures, within-between interaction, α = 0.05, 1-β = 0.80, effect size f = 0.25) indicated the study would require a sample size of 12 participants yielding an observed power of 80.1%. Descriptive data are presented as the mean and standard deviation (SD). All raw and derived data were collated and checked for outliers (where values were > 3 SD from the mean) before completing the analyses. Data modelling involved point estimation of differences between PBC and control conditions, and 90% confidence interval estimates of the uncertainty about the value of these parameters.

Data analyses were performed by SPSS version 25 (SPSS Inc., Chicago, IL, USA). A two-way repeated measure Analysis of Variance (ANOVA) [group (PBC and CON) × time point (pre-PBC, 15 min, 2 h and 3 h post-PBC)] was used to analyse salivary biomarker concentrations, CMJ performance variables and questionnaire categories across time. The significance was set at P < 0.05. Complementary standardised differences (Cohens effect size, ES) were interpreted against the following criteria: < 0.2 trivial, 0.2–0.6 small, 0.6–1.2 moderate, 1.2–2.0 large and > 2.0 large. When the magnitude of the standardised difference crossed the threshold of a small positive and small negative (-0.2 and +0.2), the change or difference was deemed unclear.

## RESULTS

### Countermovement Jump

**FIG. 2:**
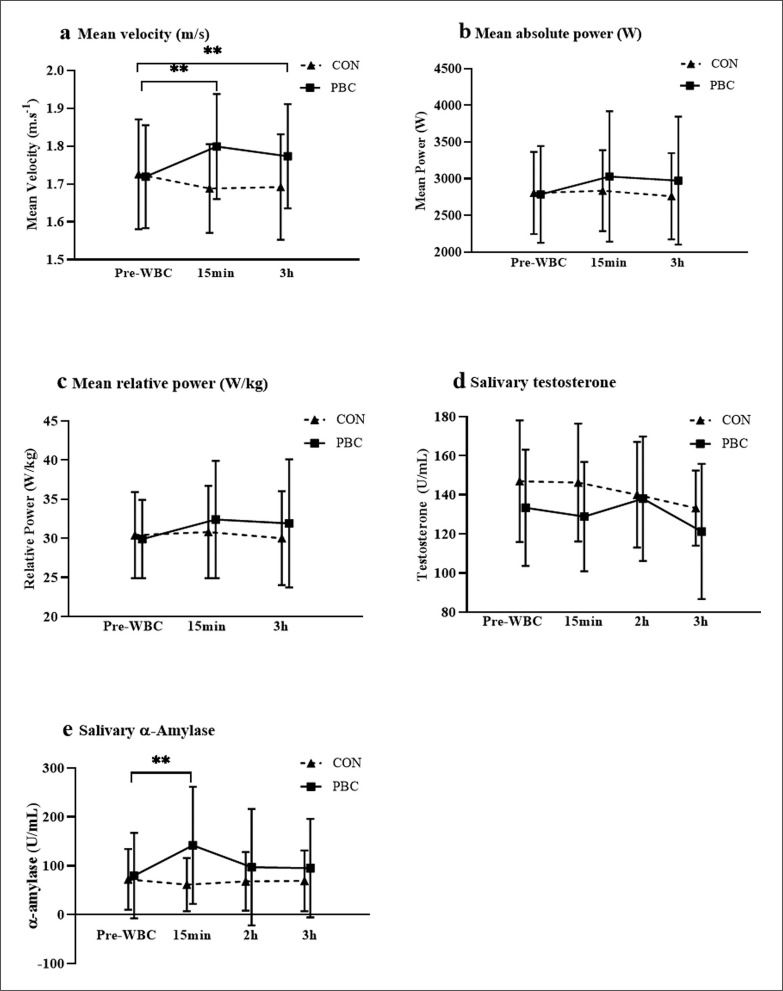
Maximal countermovement jump performance (mean ± SD) of a) velocity, b) power and c) relative power as well as saliva concentrations of d) testosterone and e) α-amylase at 3–4 time points (Baseline; 15 min post-partial-body cryotherapy; 2 h post-partial-body cryotherapy; 3 h post-partial-body cryotherapy). Substantial changes in the mean score are noted by (*)small and (**)moderate.

The increase in CMJ velocity output was greater in the PBC condition across all timepoints than the control condition (F (1, 11) = 5.154, *p* = .015). CMJ velocity was moderately higher at 15 min post-PBC (+4.7%, *p* = .006, ES = 0.81, mean ± SD, 90% confidence limits) and 3 h post-PBC (+2.3%, *p* = .019, ES = 0.62) than control (Figure 2). Absolute power (W) jump performance was not substantially altered by PBC with little difference between both the control and PBC exposure conditions across all timepoints (F(1, 11) = 2.537, *p* = .102). Although PBC exposure elicited a trend for small improvements at 15 min post-PBC (+7.6%, *p* = .408, ES = 0.22) and at 3 h post-PBC (+11.1%, *p* = .381, ES = 0.25) these were not significant. Similarly, in relative power (W/kg) there was little difference between control and PBC exposure across the 3 h period (F(1, 11) = 0.250, *p* = .627). PBC exposure elicited small but not significantly greater increases at 15 min post-PBC (7.8%, *p* = .798, ES = 0.32) and at 3 h post-PBC (5.6%, *p* = .441, ES = 0.37) than control.

### Salivary Biomarkers

Pre-exercise PBC elicited a substantial increase in the main effect of α-amylase activity compared to the control condition (F(3, 30) = 6.798, *p* = .001). The mean change in α-amylase concentration from baseline to 15 min was moderately higher after PBC (+131%, *p* = .005, ES = 0.80) compared with control (-4.2%). The increase in α-amylase concentration was higher than control at both the 2 h post-PBC (+40%, *p* = .199, ES = 0.30) and 3 h post-PBC (+60%, *p* = .116, ES = 0.27). In contrast, salivary testosterone concentration was higher in the control condition than after PBC exposure (F (1, 11) = 4.821, *p* = .05). However, the mean testosterone concentration at all timepoints was not significantly elevated in the PBC or control condition from baseline (F(3, 30) = 0.811, *p* = .497).

### Perceptual Measures

**FIG. 3:**
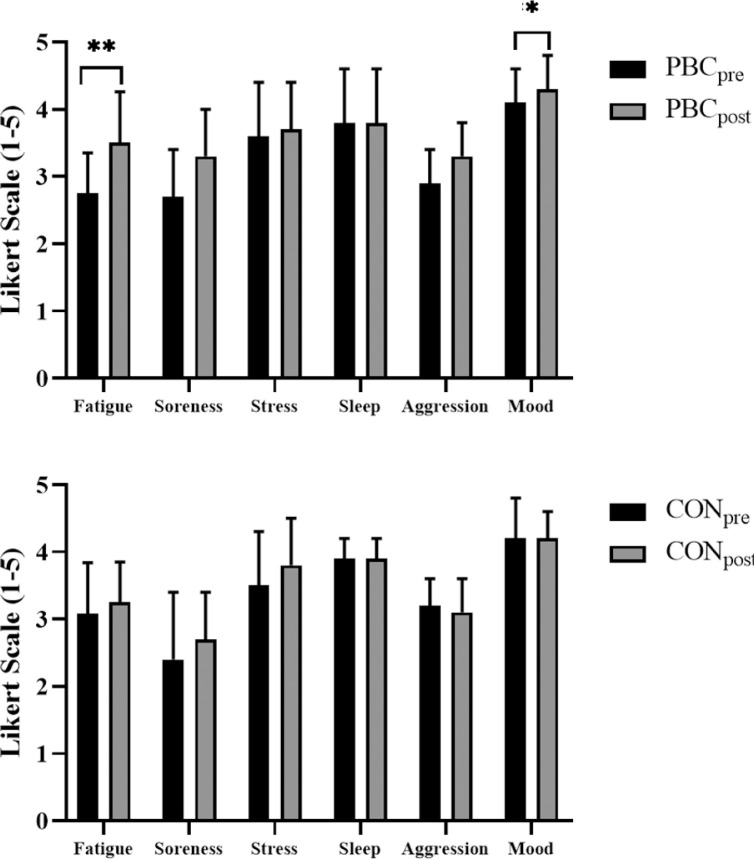
Perceptual measures recorded through the performance readiness and well-being questionnaire completed pre-post partial-body cryotherapy and control conditions. Variables are mental fatigue, muscle soreness, stress levels, sleep quality, aggression, and overall mood. Measured on a 5-point Likert scale in which a higher score is deemed optimal for performance outcomes. Any substantial changes in the mean scores are noted by (*)small and (**)moderate.

The performance readiness and overall well-being questionnaire total score (maximum score of 30 units) group mean indicated no difference between PBC and control (F(1, 11) = 0.125, *p* = .731). PBC elicits a moderately greater increase in self-reported overall performance readiness from baseline to 3 h post-PBC (F(1, 11) = 4.231, *p* = .064, ES = 0.68). Mental fatigue was similar between PBC and control conditions (F(1, 11) = 0.40, *p* = .845) but 3 h post-PBC exposure self-reported mental fatigue was moderately lower (F(1, 11) = 4.115, *p* = .067, ES = 0.79). There was no difference in the mean change in self-reported muscle soreness between PBC and control conditions (F(1, 11) = 2.850, *p* = .119) (Figure 3). However, the 3 h post-PBC scores indicated small but not significantly greater increases in muscle soreness levels (F(1, 11) = 2.099, *p* = .175, ES = 0.46). Self-reported stress indicated no significant differences in the main effects of PBC or control conditions (F(1, 11) = 0.00, *p* = 1.00). Aggression level was not substantially altered by PBC exposure with no difference between the control and PBC conditions (F(1, 11) = 0.00, *p* = 1.00). Self-reported mood was similar between PBC and control conditions (F(1, 11) = 0.133, *p* = .723). PBC exposure elicited a small improvement in mood from baseline to 3 h (F(1, 11) = 3.667, *p* = .082, ES = 0.45).

## DISCUSSION

Our results indicate that a single pre-exercise PBC exposure elicits a moderate short-term improvement in countermovement jump velocity performance 15 min after exposure, with increased performance maintained for 3 h. Small improvements in self-reported factors of performance readiness and a moderate increases in α-amylase also point to benefits of PBC for promoting improvements in athletic performance. The advantageous effect of PBC exposure, in addition to resistance-based priming, holds promise as a passive intervention during the warm-up period to better prepare athletes for competition, or to enhance training effects.

Employing pre-exercise PBC as a potential enhancer of performance in combination with resistance-based exercises has not, to our knowledge, been investigated previously. There is limited evidence on the effects of PBC exposure prior to physical performance testing. However, PBC exposure has been utilised prior to sit-in-reach tests using healthy, non-athletic participants, which elicited significant improvements in reach amplitude in men by ~2 cm and women by ~3 cm compared to the control conditions [13]. In our study using well-trained athletes, PBC yielded moderate improvements in repeated maximal effort countermovement jump velocity at 15 min and 3 h after exposure. There were small increases in absolute and relative power output 15 min after PBC compared with the control condition. However, changes in absolute and relative CMJ power were unclear 3 h after PBC exposure. The benefits of PBC-exposure are seemingly transient with a declining effect over the 3 hrs, a period that may be better suited for implementation into training sessions rather than pre-competition.

Several intrinsic factors contribute to an athlete’s eventual performance capacity and related performance outcomes [23, 24]. An athlete’s level of self-perceived stress can impair athletic performance in vertical jump performance [25]. WBC can also yield a decrease in perceived muscle soreness of -19% compared to an increase of +375% using cold-water immersion and +388% in the placebo conditions [26]. The performance readiness questionnaire results indicated substantial improvements at 3 hrs post-PBC in self-reported perceptions of mental fatigue, mood, muscle soreness and overall questionnaire score. The control condition had a small improvement in stress levels and no changes across all other measures. These perceptual benefits indicate the participants felt they were in a more prepared state of physical well-being after PBC exposure. Improved physical well-being would be useful for regular training sessions or competitions to realise these psychosocial benefits.

Several studies have reported a large variation in the effects of exposure to extreme cold on serum testosterone concentrations in athletes. Elite male soccer players exposed to 60 seconds of WBC at –60°C, then 120 seconds at –135°C after repeated maximal sprints, exhibited a 28% greater increase in testosterone concentration up to 24 h post-WBC than a control group [10]. Opposing this outcome, however, is another study which failed to observe changes in any hormonal or inflammatory biomarker following WBC exposure [27]. In our study using male athletes, the trials were completed both in early and late afternoon sessions (between 1 to 6 pm), this likely increased variability given the typical diurnal [28] and ultradian rhythm of testosterone [29], and inter-individual differences [30, 31]. The variability of testosterone response to acute PBC exposure necessitates that more research is needed before firm conclusions can be made.

Salivary α-amylase is a sensitive biomarker for both physical and psychological stress-induced influences that reflect the activity of the ANS, in particular the sympathetic nervous system (SNS) [32]. Salivary α-amylase is also correlated moderately (r = 0.30–0.66) with increases in blood catecholamines specifically, dopamine, epinephrine, and norepinephrine [16, 33]. In this study, the moderate increase from PBC exposure evident 15 min after exposure could indicate a window of opportunity to utilise the increase in SNS activity and blood catecholamines for competition. However, the practicality of implementing such a protocol so close to competition needs addressing given the current lack of readily available cryotherapy facilities in most sporting venues. Immersion in 0°C water can increased peroneal muscle SNS activity by 200 and 300% more than when submerged in 14°C water or a control condition [15]. Given the high correlation (r = 0.63) of α-amylase to venous plasma catecholamines [34], PBC exposure could be utilised to elevate SNS activation, impacting on behavioural, bioenergetic and neuromuscular systems of participants, potentially benefitting performance in heavy contact sports [35]. Using rowing athletes, α-amylase concentration was nearly two-fold higher in response to a 2000 m ergometer test in varsity athletes (105 ± 61 U/mL) than novices (56 ± 39 U/mL). The increase in sAA was linked positively with improved performance and perceived team bonding in the more experienced varsity athletes [36]. PBC exposure before high-intensity or resistance-based training could potentially elicit desirable improvements in physiology and enhance training performance.

Several limitations were identified in this study and warrant mention. Firstly, by using a resistance-based protocol to measure performance, the translation to sport-specific performance is not entirely clear. Future research needs to investigate PBC exposure on competition performance measures to verify the realistic application of PBC into a warm-up routine by athletes. Secondly, no temperature response to PBC was measured in this protocol. The parameters of skin, muscle and core temperature and the subsequent influence on performance in individuals need to be monitored and understood. Thirdly, the sample size was small, however given the participants were relatively homogenous in age, training history and sport type, our sample size was deemed sufficient to draw trustworthy conclusions. Finally, the protocol lacked any blinding due to the difficulty in masking the PBC temperature. Consequently, any beliefs held previously by the participants could have influenced perceptual responses and CMJ performance. Future studies should implement a degree of blinding to negate any of these effects.

## CONCLUSIONS

An acute session of PBC exposure holds short-term benefits to CMJ velocity performance. The relationships between psychological well-being, ANS activation and subsequent athletic performance could be positively influenced by PBC exposure during the warm-up routine. In the elite sport setting, small improvements in performance mediated by one or more interventions or strategies can be worthwhile. It appears that PBC as a passive warm-up strategy could potentially allow athletes to be in a higher state of performance readiness when used prior to competition or within training. PBC application is recommended as a complementary activity prior to active warm-up strategies rather than a sole intervention.

## Funding

No funding was received in the production of this article.

## Conflicts of Interest

The authors have no conflicts of interest to declare.
